# Reduced Serum Endostatin in Premenopausal Women with Lipedema Suggests Altered Vascular Homeostasis

**DOI:** 10.3390/diseases14070251

**Published:** 2026-07-12

**Authors:** Sally Kempa, Thomas S. Weiss, Hauke Christian Tews, Lukas Prantl, Martina Müller, Christa Buechler

**Affiliations:** 1 Department of Plastic, Hand, and Reconstructive Surgery, University Hospital Regensburg, 93053 Regensburg, Germanylukas.prantl@ukr.de (L.P.); 2 Children’s University Hospital (KUNO), University Hospital Regensburg, 93053 Regensburg, Germany; thomas.weiss@ukr.de; 3 Department of Internal Medicine I, Gastroenterology, Hepatology, Endocrinology, Rheumatology, and Infectious Diseases, University Hospital Regensburg, 93053 Regensburg, Germany; hauke.tews@ukr.de (H.C.T.); martina.mueller-schilling@ukr.de (M.M.)

**Keywords:** adipokine, hepatokine, obesity, inflammation

## Abstract

Background: Lipedema is a chronic disorder that predominantly affects women and is characterized by abnormal subcutaneous adipose tissue accumulation, pain, and vascular dysfunction. However, reliable circulating biomarkers that reflect disease-specific pathophysiology are still lacking. This study investigated serum markers associated with adipose tissue, inflammation, and angiogenesis to further elucidate the pathophysiology of lipedema. Methods: In this cross-sectional observational study, fasting serum levels of adiponectin, chemerin, lipopolysaccharide-binding protein (LBP), proprotein convertase subtilisin/kexin type 9 (PCSK9), soluble CD163 (sCD163), and soluble CD137 (sCD137)—proteins associated with obesity and inflammation—were measured in 23 premenopausal women with lipedema and 23 age-matched healthy premenopausal controls. Serum endostatin levels, an angiogenesis inhibitor, and insulin-like growth factor binding protein 2 (IGFBP2), a potent proangiogenic factor, were also assessed. Results: Patients with lipedema and obese controls had comparable body mass index, glucose, and serum lipid profiles. No significant differences were observed between groups in circulating levels of adiponectin, chemerin, LBP, PCSK9, sCD163, sCD137 and IGFBP2. In contrast, serum endostatin levels were significantly reduced in patients with lipedema (*p* = 0.038). Additional analyses demonstrated markedly higher endostatin expression in human subcutaneous adipose tissue than in the liver, suggesting that circulating endostatin levels may be related to adipose tissue mass. However, serum endostatin levels were lower in obese compared with normal-weight women (*p* < 0.001). Conclusion: Lipedema was not associated with altered circulating levels of adiponectin, chemerin, LBP, PCSK9, sCD163, sCD137 or IGFBP2. Reduced serum endostatin levels support a potential role for vascular dysfunction in the pathophysiology of lipedema.

## 1. Introduction

Lipedema is a relatively common disorder that almost exclusively affects women, with an estimated prevalence of approximately 1–10% [1,2,3,4]. It commonly begins during periods of hormonal fluctuation, such as puberty, pregnancy, or menopause, suggesting a potential hormonal, particularly estrogenic, role in its development. Since there are no specific laboratory- or imaging-based diagnostic tools, lipedema is diagnosed through a detailed patient history and clinical examination [1,3,4,5]. Diagnostic criteria for lipedema have not yet been standardized, which prevents early diagnosis in affected patients and the development of suitable therapeutic strategies [6]. Many affected individuals are obese, typically defined as having a body mass index (BMI) of at least 30 kg/m^2^, yet they do not exhibit the metabolic disturbances usually associated with adiposity. Patients with lipedema are often insulin-sensitive [7,8] and have normal glucose levels and blood pressure, suggesting that their metabolic health is better than that of age- and BMI-matched controls [1,9]. While some BMI-matched studies report a more favorable lipid profile with lower low-density lipoprotein (LDL) cholesterol and triglycerides [8,9], others have observed higher cholesterol levels in lipedema [10,11]. This heterogeneity likely reflects differences in disease stages [12] or modest sample sizes and suggests that many lipid alterations observed in lipedema may primarily reflect the underlying degree of adiposity rather than lipedema-specific pathology [13].

Obesity is linked to low-grade systemic inflammation, characterized by higher circulating levels of tumor necrosis factor (TNF), interleukin-6 (IL-6), and IL-1β [14]. As serum cytokine levels, including TNF, IL-6, and IL-1β, in patients with lipedema remained unchanged compared with those in appropriate controls [13], metaflammation appears comparable between patients with lipedema and controls. Serum C-reactive protein and serum calprotectin are markers of inflammation, released by hepatocytes or innate immune cells, respectively [15]. Both are increased in obesity [16], with C-reactive protein levels similar between patients with lipedema and obese controls. Serum calprotectin levels have not, to our knowledge, been compared between these two cohorts.

As lipedema is characterized by excessive subcutaneous adipose tissue growth, circulating adipokines, which are proteins released by adipose tissues, were also analyzed. Adiponectin, which is almost exclusively expressed by adipocytes, has beneficial metabolic and anti-inflammatory effects, and its reduced levels in obesity contribute to insulin resistance [17]. Studies have reported either elevated or normal systemic adiponectin levels in patients with lipedema relative to their respective controls [1,10,18,19]. While adiponectin was positively associated with BMI in stage 1 lipedema, this association became negative in patients with stage 2 and stage 3 disease [20]. Chemerin is another adipokine that regulates insulin sensitivity, and its systemic levels are elevated in obesity. Chemerin is also expressed in the liver, and low serum levels in patients with liver cirrhosis suggest that the liver contributes to its circulating levels [21]. Serum chemerin showed a trend toward being lower in overweight/obese patients with lipedema compared with obese, age-matched controls [19].

Proprotein convertase subtilisin/kexin type 9 (PCSK9) is a protein mostly derived from the liver. PCSK9 is also expressed in human adipocytes [22] and is induced in obesity [23,24]. Its main function is to regulate LDL receptor expression and systemic LDL levels, which increase with elevated PCSK9 levels [24]. PCSK9 also plays a role in inflammation and in the removal of lipopolysaccharide (LPS) from circulation [25,26].

Kruglikov and Scherer hypothesize that lipedema may arise from LPS within gluteofemoral white adipose tissue and suggest that future studies should place greater emphasis on investigating endotoxemia in individuals with lipedema [5]. Whether PCSK9 may contribute to the postulated endotoxemia in patients with lipedema remains unresolved. LPS-binding protein (LBP) is another protein that removes LPS from the circulation and is primarily produced in the liver. LBP serum levels are elevated in inflammatory conditions of both infectious and non-infectious origins [27,28]. In obesity, LBP is associated with inflammation, insulin resistance, and adiposity, as low LBP levels in mice reduced fat mass in female animals [29,30].

CD163 is a cell-surface receptor mainly found on monocytes and macrophages. It functions as a scavenger receptor that removes free hemoglobin and hemoglobin–haptoglobin complexes arising during intravascular hemolysis. Breakdown of these complexes generates metabolites with anti-inflammatory properties [31,32]. CD163 expression increases in response to anti-inflammatory signals, whereas pro-inflammatory cytokines reduce its expression [31]. Consequently, cells with high CD163 levels contribute significantly to the regulation of immune responses [31]. Soluble CD163 (sCD163) in the blood increases during activation of macrophages [32]. In the adipose tissue of individuals with lipedema, macrophages are predominantly M2-polarized and exhibit elevated CD163 expression [33,34].

In addition to macrophages, lymphocytes also contribute to adipose tissue regulation in obesity [35]. However, analyses of immune cell populations in lipedema adipose tissue have not identified meaningful differences in the numbers of T cells or mast cells compared with non-lipedema controls, irrespective of obesity status [11,36]. CD137 is expressed on activated T cells and, upon interaction with its ligand, amplifies immune responses. In contrast, the soluble form, sCD137, counteracts this activity and acts as an endogenous inhibitor of the bidirectional signaling pathway [37]. Elevated levels of both CD137 expression and sCD137 protein in adipose tissue have been linked to inflammation associated with obesity and related metabolic disorders [38].

The growth of adipose tissue in obesity depends on substantial vascular remodeling, particularly the formation of new blood vessels, to ensure sufficient oxygen and nutrient supply to enlarging adipocytes. During obesity, adipocytes secrete higher levels of pro-angiogenic factors, including vascular endothelial growth factor (VEGF), and endothelial cells exhibit increased expression of their receptors [39]. VEGF released from extracellular vesicles by stromal vascular cells promotes endothelial cell proliferation [40]. These cells, in turn, release vesicles to enhance M2-macrophage polarization in lipedema adipose tissue [40]. Alterations in the microvascular system have been suggested as important factors in both the onset and progression of lipedema [41]. Elevated levels of circulating VEGF have been observed in individuals with lipedema [20,42]. Recent multi-level analyses of subcutaneous adipose tissue further support the relevance of vascular and perivascular mechanisms in early-stage lipedema. Strohmeier et al. [43] reported altered gene expression in adipose tissue and stromal vascular fraction-derived cellular subpopulations, including endothelial cells and pericytes. Moreover, the secretome of lipedema-derived stromal vascular fraction cells increased endothelial leakiness in vitro and reduced VE-cadherin expression, suggesting that early lipedema may involve endothelial barrier dysfunction. These findings indicate that angiogenesis-related and endothelium-regulatory proteins may be particularly relevant to understanding the pathophysiology of lipedema. Endostatin is a C-terminal fragment of collagen XVIII and a potent anti-angiogenic protein functioning in part by blocking VEGF binding to its receptors [44]. Cathepsin S, L, and matrix metalloproteinases cleave collagen XVIII to release this 20 kDa protein. Endostatin injection was found to prevent obesity in the high-fat diet model and to lower body mass in obese mice [45,46]. These effects are related to the anti-angiogenic features of endostatin, which has been found to reduce VEGF expression in adipocytes [47]. As angiogenesis is essential for tumor growth, most studies have focused on the role of endostatin in cancer. Endostar is a modified form of endostatin, approved in China for the treatment of lung cancer, and is a potent antiangiogenic drug [44,48]. Whether serum endostatin is associated with obesity has not been finally resolved [44]. Moreover, serum endostatin levels have not been analyzed in lipedema to our knowledge.

Another protein of interest in the context of angiogenesis and obesity is insulin-like growth factor-binding protein 2 (IGFBP2), whose circulating levels are reduced in obesity [49]. IGFBP2 is abundantly expressed by preadipocytes during adipogenesis and acts as a negative regulator of adipocyte differentiation, thereby limiting adipose tissue expansion and protecting against obesity [49]. IGFBP2 regulates adipose tissue growth through multiple mechanisms, some of which depend on insulin-like growth factors (IGFs) and others of which do not. Notably, IGFBP2 also exhibits potent proangiogenic activity, including the upregulation of VEGF transcription [49]. The proangiogenic activity of IGFBP2 has not been investigated in the context of adipose tissue expansion and, based on current evidence, does not appear to compromise its protective effects against obesity.

This study aimed to define a circulating protein profile for lipedema in premenopausal women, focusing on proteins involved in adipose tissue function, inflammation, vascular regulation, and metabolic homeostasis. Fasting serum concentrations of adiponectin, chemerin, PCSK9, LBP, sCD163, sCD137, and the angiogenesis-modulating proteins endostatin and IGFBP2 were measured in 23 women with lipedema and 23 healthy, obese controls. All but adiponectin and chemerin were also analyzed in the serum of 20 normal-weight, healthy controls. The goal was to identify disease-associated serum alterations that may improve the mechanistic understanding of lipedema.

## 2. Materials and Methods

### 2.1. Patients and Controls

**Figure 1:**
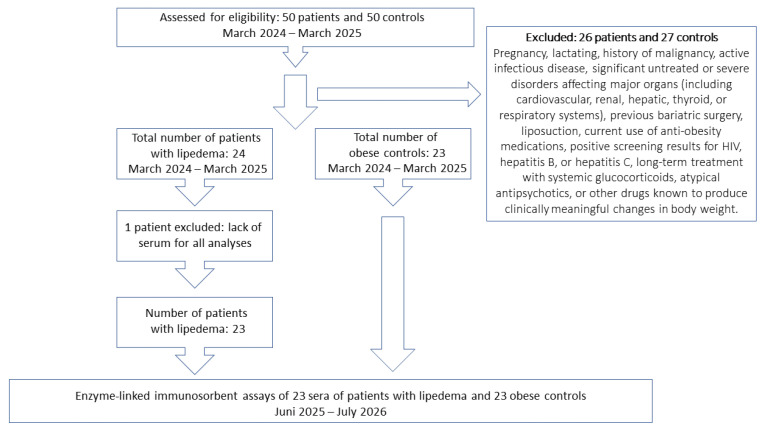
Flow chart for patient recruitment and analysis of serum proteins.

Participants were recruited between March 2024 and March 2025 consecutively during routine clinical care or through local networks (Figure 1).

Participants were excluded if they were pregnant or lactating, had a history of malignancy, had an active infectious disease, or had significant untreated or severe disorders affecting major organs, including the cardiovascular, renal, hepatic, thyroid, or respiratory systems. Additional exclusion criteria comprised previous bariatric surgery or liposuction; current use of anti-obesity medications; positive screening results for HIV, hepatitis B, or hepatitis C; and long-term treatment with systemic glucocorticoids, atypical antipsychotics, or other drugs known to produce clinically meaningful changes in body weight (Figure 1). All patients and controls, except those excluded for the reasons specified above, were included. Use of hormonal contraceptives, however, did not preclude participation in the study. Waist-to-hip ratio and BMI were self-reported by the patients and controls.

The STROBE statement [50] is shown in Table S1.

Serum from 20 healthy females, with a median age of 55 (24–64) years and BMI < 25 kg/m^2^, was also analyzed for the levels of the respective proteins. The normal-weight controls were older compared to the obese controls (*p* < 0.001).

### 2.2. ELISA

The LBP ELISA Kit was from Thermo Fisher Scientific (Hennigsdorf, Germany), the calprotectin ELISA was from Immundiagnostik AG (Bensheim, Germany), and all other ELISAs were from Bio-Techne (Wiesbaden-Nordenstadt, Germany) (Table 1). The order numbers for these tests and the serum dilution factors are given in Table 1. Standards and serum samples were assessed twice, and the average values were used in the calculations.

### 2.3. Immunoblot

Immunoblots were performed as previously described [51], with 10 μg of protein per lane. Human endostatin antibody (order number AF1098) was from Biotechne and used at a dilution of 1:2000. The GAPDH (Cell Signaling, Leiden, The Netherlands; order number 2118) and MMP9 (Bio-Techne; order number NBP1-57940SS) antibodies were diluted 1:1000. The cathepsin S antibody (Serotec, Oxford, UK, GB; order number MCA2075) was diluted 1:500.

### 2.4. Primary Cells and Tissues

Purified human preadipocytes from subcutaneous fat were obtained from BioCat (Heidelberg, Germany) and differentiated into adipocytes as suggested by the manufacturer. Subcutaneous adipose tissues and liver tissue were obtained from patients undergoing surgery for different diseases. Primary hepatocytes were isolated and cultivated as previously described [52].

### 2.5. Statistics

According to the Shapiro–Wilk test, sCD137 (*p* < 0.001), PSCK9 (*p* = 0.010), IGFBP-2 (*p* = 0.016), sCD163 (*p* = 0.014), and LBP (*p* = 0.006) were not normally distributed. Adiponectin, chemerin, and endostatin showed a normal distribution. The homogeneity of variances was present for both endostatin and chemerin, but not for adiponectin. The Mann–Whitney U test was used for all except adiponectin, chemerin, and endostatin, for which the two-sided unpaired *t*-test was used (SPSS Statistics 31.0; IBM Corp., Armonk, NY, USA, 2019). Pearson and Spearman correlation coefficients were calculated as appropriate (SPSS Statistics 31.0). Analysis of Covariance (ANCOVA) and Multivariate Analysis of Covariance were done in SPSS. A *p*-value < 0.05 was considered significant.

## 3. Results

### 3.1. Patients and Controls

This study analyzed serum proteins of 23 healthy premenopausal women and 23 premenopausal women with lipedema. Patients with lipedema had similar BMI, HbA1c, glucose, C-reactive protein, serum calprotectin, blood pressure, triglycerides, serum cholesterol, low-density lipoprotein (LDL), and high-density lipoprotein (HDL) levels as the controls. It should be noted that BMI may not accurately reflect obesity status in lipedema due to the disproportionate fat distribution [6]. As a result, reliance on BMI may partly explain variations in findings and interpretations across the existing literature [6]. Moreover, the waist-to-hip ratio is lower in lipedema due to a different fat distribution compared with obese controls (Table 2).

In the patient group, age was higher. Total fat mass was measured in only 9 controls and 19 patients, and was not compared between the two cohorts (Table 2).

### 3.2. Adiponectin and Chemerin

Serum adiponectin in patients with lipedema was 3.5 ± 1.3 µg/mL, and in the controls, 3.4 ± 1.3 µg/mL; the difference was not significant (*p* = 0.837). There is also no significant difference in serum adiponectin levels between patients and controls, F(1, 37) = 1.396, *p* = 0.245, and partial η^2^ = 0.036, whilst adjusting for BMI, age, HbA1c, glucose, HDL, and LDL. In patients, adiponectin was negatively correlated with BMI but not with total fat mass, waist-to-hip ratio, blood pressure, glucose, CRP, or calprotectin. In the controls, serum adiponectin was not associated with these measures (Table 3). A negative correlation between serum adiponectin and age was observed in the controls but was absent in the patient cohort (Table 3).

Serum chemerin levels were 126 ± 39 ng/mL in patients with lipedema and 141 ± 39 ng/mL in controls; the difference was not significant (*p* = 0.192). There is no significant difference in serum chemerin levels between patients and controls, F(1, 37) = 3.172, *p* = 0.083, and partial η^2^ = 0.079, whilst adjusting for BMI, age, HbA1c, glucose, HDL, and LDL. Here, the effect of the BMI was significant (*p* = 0.023). Serum chemerin was positively correlated with BMI in controls, and this association was nearly significant in patients with lipedema, where a significant correlation with total fat mass was observed (Table 3). Chemerin positively correlated with CRP in the controls and with diastolic blood pressure in the patients.

Serum adiponectin and chemerin levels did not correlate with triglycerides, cholesterol, or HDL in either cohort (*p* > 0.05 for all comparisons).

### 3.3. PCSK9 and LBP

**Figure 2:**
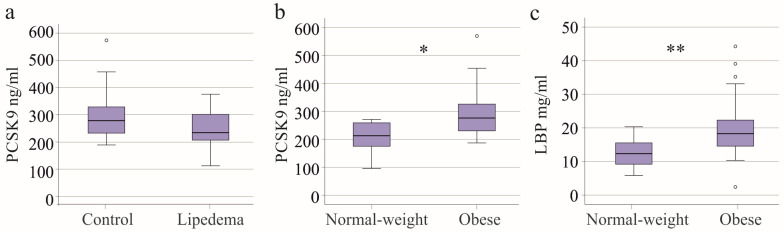
Serum PCSK9 and LBP levels in normal-weight and obese females, and PCSK9 in patients with lipedema. (**a**) Serum PCSK9 levels of obese controls and patients with lipedema; (**b**) Serum PCSK9 levels in normal-weight and obese females; (**c**) Serum LBP levels in normal-weight and obese females. * *p* < 0.05, ** *p* < 0.01. The box represents the interval containing the central 50% of the data, and the line within the box represents the median. Its lower boundary corresponds to the first quartile (Q1), while its upper boundary corresponds to the third quartile (Q3). Small circles and asterisks above or below the boxes indicate outliers.

Serum PCSK9 in patients with lipedema was 231 (109–372) ng/mL, and in controls, 275 (185–571) ng/mL; levels were similar between cohorts (*p* = 0.111, Figure 2a). There is no significant difference in serum PCSK9 levels between patients and controls, F(1, 37) = 2.790, *p* = 0.103, and partial η^2^ = 0.070, whilst adjusting for BMI, age, HbA1c, glucose, HDL, and LDL. Serum PCSK9 did not correlate with age, BMI, waist-to-hip ratio, blood pressure, glucose, CRP, or calprotectin in either cohort. PCSK9 in patients’ serum did not correlate with total fat mass (Table 4).

Currently, it has not been definitively established whether serum PCSK9 levels are higher in obese individuals. Indeed, healthy controls with a BMI ≤ 25 kg/m^2^ had lower serum PCSK9 levels than the obese controls (Figure 2b).

Serum LBP levels in patients with lipedema were 18 (8–4) mg/mL and, in controls, 18 (2–44) mg/mL; the difference was not significant (*p* = 0.717). There is no significant difference in serum LBP levels between patients and controls, F(1, 37) = 0.528, *p* = 0.472, and partial η^2^ = 0.014, whilst adjusting for BMI, age, HbA1c, glucose, HDL, and LDL. LBP was positively correlated with BMI and CRP (Table 4) in both groups and was higher in obese controls than in normal-weight controls (Figure 2c). Serum LBP levels positively correlated with fat mass in the patients (Table 4).

Serum PCSK9 and LBP levels did not correlate with triglycerides, cholesterol, HDL, or LDL in either group (*p* > 0.05 for all).

### 3.4. sCD163 and sCD137

Serum sCD163 levels were 539 (293–1239) ng/mL in patients with lipedema and 551 (233–1415) ng/mL in controls; the difference was not significant (*p* = 0.784). There is no significant difference in serum sCD163 levels between patients and controls, F(1, 37) = 0.098, *p* = 0.755, and partial η^2^ = 0.003, whilst adjusting for BMI, age, HbA1c, glucose, HDL, and LDL. Serum sCD163 positively correlated with BMI in both groups and with fat mass and systolic blood pressure in the patient cohort (Table 5). CRP positively correlated with sCD163 in patients with lipedema but not in obese controls (Table 5).

**Figure 3:**
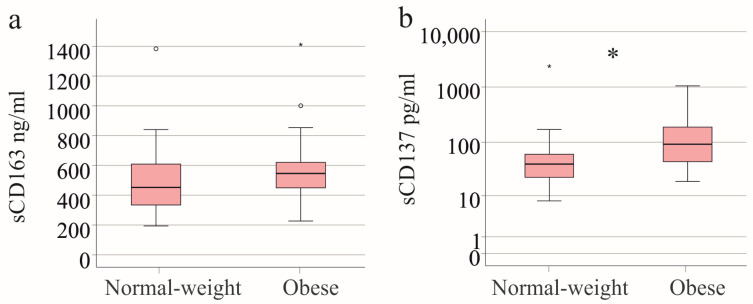
Serum sCD163 and sCD137 levels in normal-weight and obese females. (**a**) Serum sCD163 levels in normal-weight and obese females; (**b**) Serum sCD137 levels (shown on a logarithmic scale) in normal-weight and obese females. * *p* < 0.05. The box represents the interval containing the central 50% of the data, and the line within the box represents the median. Its lower boundary corresponds to the first quartile (Q1), while its upper boundary corresponds to the third quartile (Q3). Small circles and asterisks above the boxes indicate outliers.

Despite the positive correlation of sCD163 with BMI in the obese, serum levels were similar between normal-weight and obese controls (Figure 3a).

Serum sCD137 levels in patients with lipedema were 68 (24–3387) pg/mL and 93 (19–1046) pg/mL in controls; the two cohorts were similar (*p* = 0.339). There is no significant difference in serum sCD137 levels between patients and controls, F(1, 37) = 0.610, *p* = 0.440, and partial η^2^ = 0.016, whilst adjusting for BMI, age, HbA1c, glucose, HDL, and LDL. Serum sCD137 was not associated with age, BMI, waist-to-hip ratio, blood pressure, glucose, CRP, or calprotectin in both groups (Table 5). However, serum sCD137 levels were higher in obese than in normal-weight controls (Figure 3b).

Serum sCD163 and sCD137 levels did not correlate with triglycerides, cholesterol, HDL, or LDL in either group (all *p* > 0.05).

### 3.5. IGFBP2 and Endostatin

Serum IGFBP2 was 50 (30–154) ng/mL in controls and 66 (26–134) ng/mL in patients, with no significant difference between groups (*p* = 0.482). There is no significant difference in serum IGFBP2 levels between patients and controls, F(1, 37) = 0.640, *p* = 0.429, and partial η^2^ = 0.017, whilst adjusting for BMI, age, HbA1c, glucose, HDL, and LDL.

In accordance with the literature, IGFBP2 was reduced in the obese controls compared to the lean controls, who had 147 ± 79 ng/mL (*p* < 0.001). Serum IGFBP2 did not correlate with BMI, age, blood pressure, glucose, CRP, calprotectin, HDL, or LDL in either group (Table 6, *p* > 0.05 for all).

**Figure 4:**
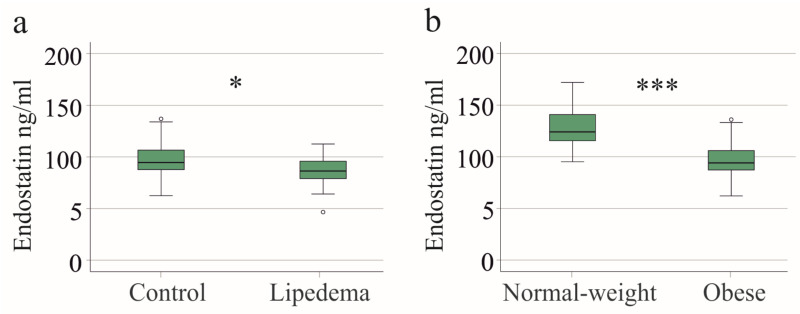
Serum endostatin levels in lipedema and levels in normal-weight and obese females. (**a**) Serum endostatin levels in patients with lipedema and obese controls; (**b**) Serum endostatin levels in normal-weight and obese female controls. * *p* < 0.05, *** *p* < 0.001. The box represents the interval containing the central 50% of the data, and the line within the box represents the median. Its lower boundary corresponds to the first quartile (Q1), while its upper boundary corresponds to the third quartile (Q3). Small circles below and above the boxes indicate outliers.

Serum endostatin was 85 ± 15 ng/mL in patients with lipedema and 96 ± 19 ng/mL in controls; it was lower in patients (*p* = 0.038) (Figure 4a). There is no significant difference in serum endostatin levels between patients and controls, F(1, 37) = 3.185, *p* = 0.083, partial η^2^ = 0.079, whilst adjusting for BMI, age, HbA1c, glucose, HDL, and LDL. Multivariate Analysis of Covariance showed that when adjusted for BMI, age, HbA1c, glucose, HDL, and LDL, none of the measured proteins differed significantly between lipedema and controls.

Because the patients were older than the controls, serum endostatin levels in patients aged <45 years were also compared. Age did not differ between the 16 lipedema patients and the 22 controls (*p* = 0.247), but endostatin was lower in the patients (*p* = 0.012).

Serum endostatin did not correlate with BMI, waist-to-hip ratio, age, blood pressure, glucose, CRP, or calprotectin in either group (Table 6). However, serum endostatin was strongly reduced in the obese (Figure 4b).

**Figure 5:**
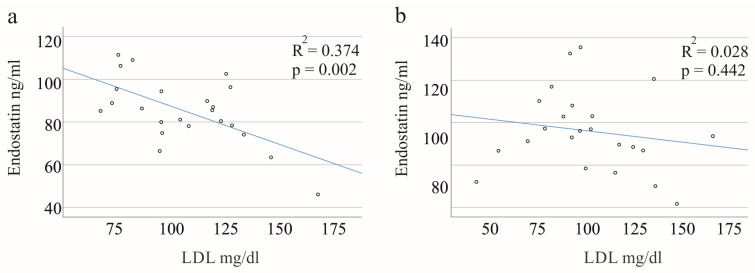
Correlation of serum endostatin levels with LDL. (**a**) Correlation of serum endostatin levels with LDL in patients with lipedema; (**b**) Correlation of serum endostatin levels with LDL in controls.

Serum endostatin levels did not correlate with triglyceride or HDL levels in either group (all *p* > 0.05). Serum endostatin was negatively correlated with LDL in patients but not in the control cohort (Figure 5a,b).

Endostatin did not correlate with adiponectin, chemerin, PCSK9, LBP, sCD137, sCD163, or IGFBP2 in the patient cohort. In the controls, there was a positive correlation of chemerin with endostatin (r = 0.453, *p* = 0.030).

### 3.6. Endostatin in Primary Human Adipose and Liver Tissue

**Figure 6:**
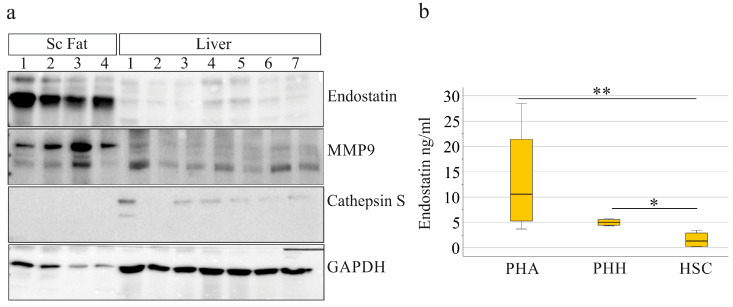
Expression of endostatin in human adipose tissue and liver. (**a**) Endostatin, MMP9, and cathepsin S in the subcutaneous adipose tissue and the liver of different human donors; (**b**) Endostatin in the supernatant of primary human adipocytes (PHAs), primary hepatocytes (PHHs), and primary hepatic stellate cells (HSCs). Levels of 10^6^ cells cultivated in 1 mL of cell culture medium are shown. * *p* < 0.05, ** *p* < 0.01.

Endostatin was reported to be primarily produced in hepatocytes but has also been detected in 3T3-L1 adipocytes [44]. Comparison of endostatin protein in the subcutaneous adipose tissue of four different donors and the liver tissues of seven donors showed that endostatin is highly abundant in fat (Figure 6a and Figure S1).

Matrix metalloproteinase 9 and cathepsin S are proteases that cleave collagen XVIII to release endostatin [44], and MMP9 was highly expressed in subcutaneous adipose tissue. Cathepsin S was detected only in the liver, suggesting it may contribute to hepatic endostatin release (Figure 6a and Figure S1).

To demonstrate that endostatin is indeed secreted by adipocytes, its levels were analyzed by ELISA in the supernatants of primary human adipocytes from 5 donors and, for comparison, in the supernatants of primary human hepatocytes from 4 donors and primary human hepatic stellate cells from 7 donors. Endostatin levels of adipocytes and hepatocytes (per 10^6^ cells) were similar, and were higher in both cell types compared to hepatic stellate cells (Figure 6b).

### 3.7. Association of the Analyzed Proteins with Lipedema Stage

In the patient cohort, 3 had stage 1, 13 had stage 2, and 6 had stage 3 lipedema. The stage of 1 patient was not documented (Table 2). BMI and CRP were positively correlated with lipedema stages (Table 7). All of the proteins analyzed, as well as HDL and LDL, were not related to the lipedema stage (*p* > 0.05 for all).

## 4. Discussion

Together, these results show lower circulating endostatin levels in lipedema and support further investigation of angiogenic and microvascular pathways as potential targets for disease diagnosis and therapeutic development.

This study demonstrates that premenopausal women with lipedema have lower circulating levels of the angiogenesis-modulating protein endostatin compared with BMI-matched controls. In contrast, serum concentrations of adiponectin, chemerin, PCSK9, LBP, sCD163, sCD137, and IGFBP2 were comparable between groups. It should be noted that our cohorts were small, which may have prevented the identification of significant differences. Nevertheless, these findings suggest that lipedema is not characterized by broad systemic alterations in the inflammatory, metabolic, or adipokine-related markers analyzed here, but rather point toward altered vascular homeostasis and angiogenic regulation as potentially relevant components of disease pathophysiology.

Importantly, the lipedema and control cohorts were well matched for key metabolic and cardiovascular variables, including BMI, blood pressure, HbA1c, LDL cholesterol, HDL cholesterol, and triglycerides. Although controls were slightly younger, differences were small and are unlikely to have materially confounded the main findings. The finding of reduced endostatin levels in patients with lipedema was confirmed in an age-matched subgroup, demonstrating that the observed difference was not attributable to age. Correlation analyses showed that adiponectin was negatively associated with age in controls; however, this association is unlikely to have masked relevant group differences.

Adjusting for age, BMI, HbA1c, glucose, HDL, and LDL revealed that there was no significant difference in adiponectin, chemerin, PCSK9, LBP, sCD163, sCD137, and IGFBP2 serum levels between patients and controls.

Serum adiponectin levels of patients with lipedema were normal, consistent with previous findings [18,20]. In patients with lipedema, serum adiponectin levels were negatively correlated with BMI, in accordance with lower levels in the obese [17]. This association did not exist in the obese controls. A combined analysis of data from healthy individuals was conducted to assess whether obesity, independent of accompanying metabolic abnormalities, influenced circulating adipokine concentrations. As expected, plasma leptin levels increased in association with BMI [53]. In contrast, no significant relationship was observed between BMI and circulating adiponectin levels among healthy adults across normal-weight to obese categories. This study included 4852 subjects and suggested that factors besides obesity affect plasma adiponectin levels [53]. Chemerin positively correlated with BMI in both groups and with fat mass in patients with lipedema. Moreover, LBP, which is induced in obesity [54], was positively correlated with BMI in both cohorts. These findings indicate that, overall, the relationships between circulating protein levels and BMI/obesity remain largely preserved in individuals with lipedema. However, BMI may not be an optimal indicator of obesity in this population because lipedema is characterized by a distinct, disproportionate pattern of adipose tissue accumulation [6]. Consequently, the use of BMI as a measure of adiposity could contribute to inconsistencies in reported results and their interpretation across studies [6]. In addition, individuals with lipedema exhibit a lower waist-to-hip ratio than obese controls, reflecting their unique body fat distribution pattern. Consequently, the adjustment for waist-to-hip ratio may also be subject to confounding.

LBP is an acute-phase protein that binds LPS and is used as a marker of gut permeability [55], and normal levels in patients with lipedema argue against increased bacterial translocation from the gut. Moreover, PCSK9 also plays a role in LPS removal [23], but serum levels did not differ in lipedema.

Systemic sCD163 is a long-lasting marker of inflammation [32]. Higher levels of sCD163 in circulation have been associated with activation of tissue macrophages, including hepatic Kupffer cells and adipose tissue macrophages [56]. Increased systemic levels in the obese and associations with insulin resistance and CRP have been consistently reported [56,57]. In patients with lipedema and controls, serum sCD163 positively correlated with BMI, while associations with CRP were only observed in the patients. However, our normal-weight cohort was too small to detect a significantly higher sCD163 level compared with obese individuals. Correlations with systolic blood pressure were weak and require confirmation in larger populations. As sCD163 levels in patients with lipedema and controls were similar, the higher expression of CD163 in lipedema adipose tissue [1] does not translate into higher sCD163 levels in the blood.

sCD137 is an antagonist for T-cell activation [37], but its levels were similar between patients with lipedema and controls. Levels of sCD137 did not correlate with BMI, fasting glucose, or CRP, but were higher in the obese than in the normal-weight controls. Thus, the association of blood sCD137 with higher BMI is preserved in lipedema. No significant differences in calprotectin concentrations were observed between the lipedema and control groups. Given that calprotectin is released by cells of the innate immune system and serves as an indicator of inflammation [58], these results further suggest that lipedema is not associated with a generalized systemic inflammatory response.

Among all proteins evaluated, endostatin was the only marker that differed significantly between patients and controls, with lower circulating levels detected in individuals with lipedema. Correlation analyses further demonstrated positive associations of endostatin with LDL cholesterol in the lipedema group, whereas these relationships were not observed in control subjects. Because LDL concentrations were comparable between patients and obese controls, the biological and pathophysiological significance of these correlations remains uncertain. Adjusting for age, BMI, HbA1c, glucose, HDL, and LDL revealed no significant difference in serum endostatin levels between patients and controls. This indicates that endostatin levels are associated with these covariates. However, after adjusting for BMI, HbA1c, and LDL, there was a significant difference in serum endostatin levels between patients and controls (*p* = 0.044). Further study is needed to evaluate the association between these covariates and serum endostatin levels in both controls and patients.

Endostatin levels also declined in obesity and were lower in the obese than in the normal-weight controls. In patients with acute myocardial infarction, the obese group, which consisted of males and females, also had reduced systemic endostatin levels [59]. Nevertheless, the validity of this comparison is constrained by the poor clinical condition of the patients included in the study. Silha et al. reported higher endostatin levels in obese females, but this difference was not significant in males [60]. Like our investigation, this study evaluated fasting blood samples [60]. Consistent with established findings, individuals with obesity exhibited higher leptin levels than controls [60]. Endostatin concentrations were determined in serum using a commercially available ELISA assay [60], employing an approach comparable to that used in the present study. Currently, no clear explanation can be provided for the inconsistencies in endostatin concentrations among individuals with obesity.

Endostatin was found to inhibit adipogenesis [61], and lower serum levels in obese patients are consistent with excess adipose tissue. Endostatin is an angiogenesis inhibitor primarily derived from hepatocytes [44]. However, lower levels in the obese suggest that adipose tissue may also affect serum endostatin levels. Comparison of endostatin protein levels in human subcutaneous fat and the liver showed that subcutaneous adipose tissue has high endostatin levels. Endostatin is also released by primary human adipocytes, suggesting that adipose tissues can contribute to its systemic levels. As levels decline in obesity—a phenomenon also observed for adiponectin [51]—studies examining its expression in obese adipose tissue are warranted. Previous studies have reported increased endothelial cell proliferation in lipedema-associated adipose tissue, which may facilitate tissue growth and remodeling [62]. In this context, lower circulating endostatin concentrations could support these changes by reducing anti-angiogenic activity.

Notably, VEGF, whose activity is blocked by endostatin, is elevated in lipedema [20,42,44], further supporting the suggestion that angiogenesis is enhanced in patients with lipedema. In addition to altered circulating angiogenesis-related proteins, imaging-based studies may provide further evidence of local microvascular abnormalities in lipedema [13,63].

Human umbilical vein endothelial cells cultured in conditioned media from either healthy or lipedema adipocytes showed higher VEGF expression in the latter experimental setting [64]. In addition, recent multi-level analyses of early-stage lipedema adipose tissue demonstrated the relevance of perivascular cell populations and endothelial barrier dysfunction [43]. In that study, the secretome of lipedema-derived stromal vascular fraction cells increased endothelial permeability in vitro and was associated with reduced VE-cadherin expression. Endostatin was found to suppress VEGF produced in adipose tissue [44], and lower endostatin levels are principally in accordance with higher VEGF. Thus, the reduced circulating endostatin levels observed in the present study may complement tissue- and cell-based evidence of adipose tissue–vascular dysregulation in lipedema.

IGFBP2 is a potent proangiogenic factor, yet its circulating concentrations were comparable between individuals with lipedema and healthy controls [49]. In addition, IGFBP2 has been reported to suppress adipogenesis and is typically found at lower levels in obesity, supporting its proposed antiadipogenic role. Based on the present findings, there is no evidence of significant involvement of IGFBP2 in the pathophysiology of lipedema.

Notably, except for BMI and CRP, none of the proteins examined in this study were associated with lipedema stage. This observation may indicate that alterations in circulating endostatin levels occur during the early phases of the disease rather than reflecting disease progression. However, given the limited sample size of the present cohort, this interpretation should be considered preliminary and requires validation in larger studies.

Several limitations should be considered when interpreting the findings of this study. First, the relatively small sample size may limit the generalizability of the results. In addition, BMI and waist-to-hip ratio were calculated from self-reported measurements in both patients and controls, introducing the possibility of reporting bias. Hormonal status was not assessed and therefore could not be accounted for in the analyses. Furthermore, the biological factors governing circulating endostatin concentrations have not yet been fully characterized, and the mechanisms responsible for altered endostatin levels in obesity and lipedema remain to be elucidated.

Despite these limitations, the study has notable strengths. The patient and control groups were carefully matched, and both cohorts exhibited largely normal lipid and glucose profiles, reducing potential metabolic confounding. The study population was restricted to premenopausal women, and stringent exclusion criteria were applied, thereby contributing to cohort homogeneity and strengthening the validity of the comparisons.

## 5. Conclusions

Together, these findings suggest that lipedema may be better understood not as a disorder of generalized systemic inflammation, but as a disease involving early dysregulation of the adipose tissue–vascular interface, with endostatin representing a promising translational biomarker for future investigation.

## Figures and Tables

**Table 1:** Order number, company name, and dilution of serum for the ELISA tests used in this study.

Protein	Company	Order Number	Dilution of Serum
Adiponectin	Bio-Techne	DY1065	1:5000
Calprotectin	Immundiagnostik AG	K 6935	1:100
Chemerin	Bio-Techne	DY2324	1:250
Endostatin	Bio-Techne	DY1098	1:100
IGFBP2	Bio-Techne	DY674	1:100
LBP	Thermo Fisher Scientific	EH297RB	1:1000
sCD163	Bio-Techne	DC1630	1:10
sCD137	Bio-Techne	DY838	1:2

**Table 2:** Characteristics of the 23 female controls and the 23 females with lipedema. The mean ± standard deviation for normally distributed measures and the median (minimum–maximum) values are listed for non-normally distributed values. If values were not obtained from the entire cohort, the respective numbers are given in brackets.

	Lipedema (23 Females)	Control (23 Females)	*p*-Value
Age, years	41 ± 6	36 ± 7	0.016
BMI, kg/m^2^	37.2 ± 8.1	34.4 ± 7.6	
Waist-to-Hip Ratio	0.76 ± 0.08 (21 patients)	0.86 ± 0.07 (21 controls)	<0.001
Total fat mass, kg	42 (19–73) (19 patients)	33 (15–46) (9 controls)	
Lipedema Stage			
1	3		
2	13		
3	6		
Not documented	1		
HbA1c, %	5.5 (5.0–6.4)	5.5 (5.1–8.3)	
Glucose, mg/dL	81 (30–94) (22 patients)	85 (67–222)	
C-reactive protein, mg/L	3.1 (0.1–48.5)	2.4 (0.1–36.8)	
Calprotectin, ng/mL	1113 (469–2272)	1205 (461–2107)	
Systolic BP, mm Hg	129 (97–180) (22 patients)	129 (110–180) (22 controls)	
Diastolic BP, mm Hg	83 (49–142) (22 patients	80 (68–115) (22 controls)	
Triglycerides, mg/dL	116 (54–265)	94 (39–342)	
Cholesterol, mg/dL	189 ± 28	182 ± 30	
LDL, mg/dL	107 ± 25	104 ± 30	
HDL, mg/dL	52 ± 10	54 ± 10	

**Table 3:** Correlation coefficients and *p*-values for the associations of adiponectin and chemerin with age, body mass index (BMI), blood pressure (BP), total fat mass (in lipedema), glucose, C-reactive protein (CRP), and calprotectin. Significant correlations are in bold. (Measures not documented for the entire cohort: Waist-to-Hip Ratio: 21 patients and 21 controls; fat mass: 19 patients and 9 controls; blood pressure: 22 patients and 22 controls; glucose: 22 patients.)

Measures	Statistics	Adiponectin		Chemerin	
		Patients	Controls	Patients	Controls
Age	r *p*	0.106 0.629	−0.525 0.010	−0.130 0.554	0.158 0.472
BMI	r *p*	−0.479 0.021	−0.144 0.511	0.414 0.050	0.468 0.024
Waist-to-Hip Ratio	r *p*	−0.228 0.320	−0.009 0.968	0.266 0.243	0.144 0.535
Syst BP	r *p*	−0.481 0.032	−0.191 0.394	0.262 0.239	0.043 0.849
Diastolic BP	r *p*	−0.371 0.089	−0.057 0.800	0.428 0.047	−0.133 0.556
Fat mass	r *p*	−0.325 0.174		0.488 0.034	
Glucose	r *p*	0.084 0.710	−0.257 0.236	0.062 0.783	0.308 0.160
CRP	r *p*	−0.180 0.410	−0.217 0.321	0.275 0.204	0.556 0.006
Calprotectin	r *p*	0.218 0.318	−0.341 0.112	0.019 0.937	0.161 0.464

**Table 4:** Correlation coefficients and *p*-values for the associations of PCSK9 and LBP with age, body mass index (BMI), blood pressure (BP), total fat mass (in lipedema), glucose, C-reactive protein (CRP), and calprotectin. Significant correlations are in bold. (Measures not documented for the entire cohort: Waist-to-Hip Ratio: 21 patients and 21 controls; fat mass: 19 patients and 9 controls; blood pressure: 22 patients and 22 controls; glucose: 22 patients.)

Measures	Statistics	PCSK9		LBP	
		Patients	Controls	Patients	Controls
Age	r *p*	0.015 0.946	−0.007 0.975	−0.364 0.088	−0.072 0.744
BMI	r *p*	−0.007 0.975	−0.025 0.911	0.507 0.014	0.491 0.017
Waist-to-Hip Ratio	r *p*	0.211 0.358	0.161 0.485	0.176 0.446	−0.011 0.962
Syst BP	r *p*	0.061 0.787	0.033 0.883	0.165 0.463	0.276 0.214
Diast BP	r *p*	0.061 0.787	0.210 0.347	0.214 0.338	0.403 0.062
Fat mass	r *p*	−0.075 0.759		0.479 0.038	
Glucose	r *p*	0.266 0.232	0.265 0.222	−0.349 0.112	0.570 0.005
CRP	r *p*	−0.060 0.784	0.194 0.375	0.693 <0.001	0.751 <0.001
Calprotectin	r *p*	0.124 0.574	−0.185 0.422	0.042 0.847	−0.073 0.742

**Table 5:** Correlation coefficients and *p*-values for the associations of sCD163 and sCD137 with age, body mass index (BMI), blood pressure (BP), total fat mass (in lipedema), glucose, C-reactive protein (CRP), and calprotectin. Significant correlations are in bold. (Measures not documented for the entire cohort: Waist-to-Hip Ratio: 21 patients and 21 controls; fat mass: 19 patients and 9 controls; blood pressure: 22 patients and 22 controls; glucose: 22 patients.)

Measures	Statistics	sCD163		sCD137	
		Patients	Controls	Patients	Controls
Age	r *p*	−0.161 0.464	0.248 0.253	−0.353 0.098	0.255 0.240
BMI	r *p*	0.791 <0.001	0.438 0.037	−0.143 0.514	−0.369 0.084
Waist-to-Hip Ratio	r *p*	0.390 0.081	0.102 0.660	0.050 0.829	−0.218 0.342
Syst BP	r *p*	0.483 0.023	0.210 0.347	−0.150 0.505	−0.132 0.557
Diast BP	r *p*	0.397 0.068	0.064 0.778	0.131 0.562	−0.018 0.938
Fat mass	r *p*	0.705 <0.001		−0.254 0.293	
Glucose	r *p*	0.047 0.836	0.104 0.638	0.250 0.261	−0.327 0.127
CRP	r *p*	0.738 <0.001	0.351 0.101	0.190 0.385	−0.383 0.071
Calprotectin	r *p*	−0.125 0.571	0.036 0.870	−0.157 0.474	0.197 0.367

**Table 6:** Correlation coefficients and *p*-values for the associations of IGFBP2 and endostatin with body mass index (BMI), age, blood pressure (BP), total fat mass (in lipedema), glucose, C-reactive protein (CRP), and calprotectin. (Measures not documented for the entire cohort: Waist-to-Hip Ratio: 21 patients and 21 controls; fat mass: 19 patients and 9 controls; blood pressure: 22 patients and 22 controls; glucose: 22 patients.)

Measures	Statistics	IGFBP2		Endostatin	
		Patients	Controls	Patients	Controls
Age	r *p*	0.051 0.817	0.043 0.847	0.319 0.138	−0.025 0.909
BMI	r *p*	−0.084 0.705	−0.035 0.876	0.140 0.524	−0.005 0.983
Waist-to-Hip Ratio	r *p*	0.072 0.757	−0.398 0.074	−0.081 0.726	−0.182 0.430
Syst BP	r *p*	−0.163 0.468	0.175 0.436	0.093 0.680	−0.099 0.661
Diast BP	r *p*	−0.306 0.166	−0.111 0.621	0.006 0.998	−0.072 0.751
Fat mass	r *p*	0.037 0.881		0.000 0.982	
Glucose	r *p*	−0.214 0.340	0.122 0.580	0.2337 0.126	0.012 0.958
CRP	r *p*	0.008 0.970	0.006 0.977	0.017 0.937	0.063 0.773
Calprotectin	r *p*	0.148 0.500	0.162 0.460	0.315 0.143	−0.174 0.426

**Table 7:** Correlation coefficients and *p*-values for the associations of the analyzed proteins, BMI, waist-to-hip ratio, CRP, and calprotectin with lipedema stage. (Measures not documented for the entire cohort: Waist-to-Hip Ratio: 21 patients and 21 controls.)

Measures	Lipedema Stage	
Age	r *p*	−0.012 0.957
BMI	r *p*	0.572 0.005
Waist-to-Hip Ratio	r *p*	−0.351 0.119
HbA1c	r *p*	−0.261 0.241
Adiponectin	r *p*	−0.235 0.291
Chemerin	r *p*	0.204 0.363
PCSK9	r *p*	−0.317 0.193
LBP	r *p*	0.419 0.151
sCD163	r *p*	0.294 0.185
sCD137	r *p*	−0.311 0.159
IGFBP2	r *p*	0.224 0.317
Endostatin	r *p*	0.011 0.960
CRP	r *p*	0.551 0.008
Calprotectin	r *p*	−0.087 0.699

## Data Availability

Data are shown in the manuscript, and datasets underlying the study are included as Supplementary Materials. Original Western blot images are also provided.

## Supplementary Materials

The following supporting information can be downloaded at https://www.mdpi.com/article/10.3390/diseases14070251/s1, Table S1: STROBE Statement. Table S2: Data on individual patients and controls. Figure S1: Original Western blots.

## Abbreviations

The following abbreviations are used in this manuscript:

BMI	Body mass index
CRP	C-reactive protein
HDL	High-density lipoprotein
IGFBP2	Insulin-like growth factor binding protein 2
Interleukin	IL
LPS	Lipopolysaccharide
LBP	Lipopolysaccharide-binding protein
LDL	Low-density lipoprotein
PCSK9	Proprotein convertase subtilisin/kexin type 9
sCD137	Soluble CD137
sCD163	Soluble CD163
TNF	Tumor necrosis factor
