# Supplementary material for: Reduced Serum Endostatin in Premenopausal Women with Lipedema Suggests Altered Vascular Homeostasis
Source: Diseases. 2026 Jul 12;14(7):251. doi: 10.3390/diseases14070251 (PMC13409243; doi:10.3390/diseases14070251)
Supplement: Supplementary file 1 [file diseases-14-00251-s001.zip › diseases-4385896-supplementary.pdf]

Table S1: STROBE Statement

	Item No	Recommendation	
**Title and abstract**	1	(*a*) Indicate the study's design with a commonly used term in the title or the abstract	Abstract
		(*b*) Provide in the abstract an informative and balanced summary of what was done and what was found	Page 1
**Introduction**			
Background/rationale	2	Explain the scientific background and rationale for the investigation being reported	Page 1 – 4
Objectives	3	State specific objectives, including any prespecified hypotheses	Page 4
**Methods**			
Study design	4	Present key elements of study design early in the paper	Abstract<br>2.1.<br>Figure 1
Setting	5	Describe the setting, locations, and relevant dates, including periods of recruitment, exposure, follow-up, and data collection	2.1.<br>Figure 1
Participants	6	(*a*) *Cohort study*—Give the eligibility criteria, and the sources and methods of selection of participants. Describe methods of follow-up<br>*Case-control study*—Give the eligibility criteria, and the sources and methods of case ascertainment and control selection. Give the rationale for the choice of cases and controls<br>*Cross-sectional study*—Give the eligibility criteria, and the sources and methods of selection of participants	2.1.<br>Figure 1
		(*b*) *Cohort study*—For matched studies, give matching criteria and number of exposed and unexposed<br>*Case-control study*—For matched studies, give matching criteria and the number of controls per case	Not applicable
Variables	7	Clearly define all outcomes, exposures, predictors, potential confounders, and effect modifiers. Give diagnostic criteria, if applicable	2.1.<br>Table 2
Data sources/ measurement	8*	For each variable of interest, give sources of data and details of methods of assessment (measurement). Describe comparability of	Materials and Mehods

		assessment methods if there is more than one group	
Bias	9	Describe any efforts to address potential sources of bias	*Page 11. Age-adjusted analysis*
Study size	10	Explain how the study size was arrived at	Figure 1
Quantitative variables	11	Explain how quantitative variables were handled in the analyses. If applicable, describe which groupings were chosen and why	2.5.
Statistical methods	12	(*a*) Describe all statistical methods, including those used to control for confounding	2.5.
		(*b*) Describe any methods used to examine subgroups and interactions	2.5.
		(*c*) Explain how missing data were addressed	Not applicable
		(*d*) *Cohort study*—If applicable, explain how loss to follow-up was addressed<br>*Case-control study*—If applicable, explain how matching of cases and controls was addressed<br>*Cross-sectional study*—If applicable, describe analytical methods taking account of sampling strategy	Not applicable
		(*e*) Describe any sensitivity analyses	ANCOVA/MANCOVA

Continued on next page

**Results**			
Participants	13*	(a) Report numbers of individuals at each stage of study—eg numbers potentially eligible, examined for eligibility, confirmed eligible, included in the study, completing follow-up, and analysed	All Table legends
		(b) Give reasons for non-participation at each stage	Not applicable
		(c) Consider use of a flow diagram	Figure 1
Descriptive data	14*	(a) Give characteristics of study participants (eg demographic, clinical, social) and information on exposures and potential confounders	2.1. and Table 2
		(b) Indicate number of participants with missing data for each variable of interest	All Table legends
		(c) *Cohort study*—Summarise follow-up time (eg, average and total amount)	Not applicable
Outcome data	15*	*Cohort study*—Report numbers of outcome events or summary measures over time	
		Case-control study—Report numbers in each exposure category, or summary measures of exposure	
		Cross-sectional study—Report numbers of outcome events or summary measures	*Result part*
Main results	16	(*a*) Give unadjusted estimates and, if applicable, confounder-adjusted estimates and their precision (eg, 95% confidence interval). Make clear which confounders were adjusted for and why they were included	ANCOVA for all analyzed proteins
		(*b*) Report category boundaries when continuous variables were categorized	
		(*c*) If relevant, consider translating estimates of relative risk into absolute risk for a meaningful time period	
Other analyses	17	Report other analyses done—eg analyses of subgroups and interactions, and sensitivity analyses	Page 10<br>ANCOVA for all analyzed proteins
**Discussion**			
Key results	18	Summarise key results with reference to study objectives	Page 13
Limitations	19	Discuss limitations of the study, taking into account sources of potential bias or imprecision. Discuss both direction and magnitude of any potential bias	End of discussion
Interpretation	20	Give a cautious overall interpretation of results considering objectives, limitations, multiplicity of analyses, results from similar studies, and other relevant evidence	Conclusion
Generalisability	21	Discuss the generalisability (external validity) of the study results	Page 14
**Other information**			
Funding	22	Give the source of funding and the role of the funders for the present study and, if applicable, for the original study on which the present article is based	Page 15

Table S2: Data on individual patients and controls.

Number	Age. years	BMI. kg/m2	Waist-to-Hip Ratio	Total fat mass. g	Lipedema Stage	1 Lip 2 Control	HbA1c. %	Glucose. mg/dl	CRP. mg/l	Calprotectin. ng/ml
1	47.00	38.70	.97	44726.00	2	1.00	6.4		1.80	1621.80
2	44.00	26.00		22082.00		2.00	5.2	67.00	.70	1621.80
3	44.00	40.70	.85			2.00	6.1	94.00	19.30	1801.00
4	43.00	20.20	.68	15347.00		2.00	5.4	75.00	.10	1801.00
5	35.00	33.60	.73	38127.00	2	1.00	5.8	80.00	2.60	1707.80
6	34.00	32.40	.87			2.00	5.5	96.00	1.20	1434.30
7	38.00	54.10				2.00	8.3	222.00	36.80	1452.10
8	37.00	27.50	.87	32532.00		2.00	5.4	87.00	1.70	2106.70
9	40.00	30.90	.62	35304.00	3	1.00	5	84.00	.80	879.50
10	37.00	49.10	.95			2.00	5.5	78.00	6.30	1515.20
11	32.00	46.70	.81	71136.00	2	1.00	6.3	86.00	14.30	675.00
12	37.00	33.52	.85			2.00	5.4	86.00	1.10	1036.10
13	39.00	41.90	.78	54138.00	2	1.00	5.4	87.00	11.70	1488.00
14	30.00	25.70	.85			2.00	5.2	75.00	.70	1412.30
15	40.00	28.70	.78	31361.00		2.00	5.1	78.00	.10	879.50
16	37.00	28.10	.78	33560.00	1	1.00	5.7	83.00	.10	858.70
17	51.00	41.30	.85	45046.00	2	1.00	5.9	92.00	3.10	929.20
18	39.00	37.10	.93			2.00	5.8	111.00	3.10	1156.60
19	37.00	27.00	.87	31161.00		2.00	5.9	100.00	2.20	1204.90
20	43.00	35.20			2	1.00	5.4	62.00	4.80	1113.10
21	47.00	35.60	.78	48650.00	2	1.00	5.5	30.00	.80	1846.00
22	44.00	35.40	.80			2.00	6.8	101.00	11.60	1776.20
23	42.00	29.80	.74	34005.00	1	1.00	5.7	94.00	3.20	1124.90
24	31.00	52.00				1.00	6	63.00	48.50	1717.50
25	42.00	31.20	.72	43468.00	2	1.00	5.3	77.00	.60	1746.70
26	39.00	33.60	.83			2.00	5.6	86.00	3.40	750.10
27	44.00	39.00	.97	45657.00		2.00	5.5	82.00	1.00	976.60
28	30.00	34.40	.69	40708.00	3	1.00	5.1	66.00	10.90	1533.50
29	42.00	34.00	.82	41658.00	2	1.00	5.3	82.00	4.10	2271.80
30	31.00	37.20	.96			2.00	5.3	104.00	17.20	828.00
31	35.00	33.40	.96	35524.00		2.00	5.6	82.00	1.00	930.50
32	38.00	38.80	.70		3	1.00	5.5	73.00	17.80	687.50
33	48.00	29.80	.72	41073.00	2	1.00	5.4	81.00	1.20	918.40
34	47.00	39.10	.85			2.00	5.9	87.00	7.00	1907.00
35	28.00	31.10	.83	41648.00		2.00	5.4	85.00	3.30	564.90
36	37.00	20.40	.73	18822.00	1	1.00	5.4	81.00	.10	824.40
37	50.00	40.00	.76	48358.00	3	1.00	5.9	83.00	6.40	965.40
38	19.00	29.90	.82			2.00	5.3	78.00	3.20	1213.00
39	43.00	43.40	.81	50681.00	2	1.00	5.6	80.00	1.60	468.80
40	34.00	55.50	.77	73146.00	3	1.00	5.6	78.00	13.30	556.30
41	39.00	34.60	.74	42250.00	2	1.00	5.3	86.00	.60	2031.90
42	25.00	36.70	.93			2.00	5.5	80.00	2.20	748.00
43	26.00	33.10	.81	35522.00		2.00	5.3	82.00	16.20	1043.70
44	47.00	46.20	.63		3	1.00	5.3	79.00	6.40	1275.80
45	28.00	41.10	.76			2.00	5.5	76.00	2.40	461.00
46	46.00	33.20	.72	37936.00	2	1.00	5.8	87.00	1.00	1028.60

Number	Systol BP	Diastol BP	Apn μg/ml	Chemerin ng/ml	PCSK9 ng/ml	LBP mg/ml	sCD163 ng/ml	sCD137 pg/ml	IGFBP2 ng/ml
1	136.00	83.00	1.95	97.50	149.33	21	706.86	28.02	154.32
2	115.00	77.00	1.72	147.05	226.38	12	606.91	638.48	87.76
3	153.00	96.00	2.13	215.71	264.40	44	1004.49	191.13	57.38
4	110.00	75.00	1.57	117.98	204.00	10	607.32	1046.36	65.18
5	117.00	69.00	1.52	97.93	294.75	16	613.87	56.60	68.07
6	145.00	90.00	2.52	85.99	200.19	14	232.56	272.25	48.31
7	180.00	80.00	1.54	190.24	192.21	22	1414.81	91.87	94.77
8	121.00	77.00	3.74	108.53	326.77	17	441.22	36.99	89.03
9	106.00	75.00	2.58	103.51	127.16	12	372.74	101.26	40.22
10	150.00	80.00	3.20	134.53	275.08	12	586.38	120.47	30.19
11	148.00	91.00	2.90	150.26	199.20	27	971.15	42.16	36.16
12			5.05	161.17	327.43	22	314.45	368.46	133.53
13			2.62	161.17	251.91	20	806.59	167.76	50.69
14	150.00	80.00	5.01	69.48	185.34	2	447.77	160.14	88.98
15	127.00	81.00	2.63	105.28	416.08	13	477.46	130.81	45.43
16	113.00	85.00	3.35	107.12	304.91	18	337.88	112.31	37.19
17	180.00	108.00	.97	151.03	244.51	15	548.56	40.50	64.19
18	141.00	86.00	3.07	141.18	281.21	18	777.67	20.35	59.04
19	113.00	68.00	3.91	130.49	454.23	15	460.95	92.68	30.12
20	108.00	67.00	4.35	180.68	206.77	25	453.08	62.13	90.09
21	160.00	77.00	2.78	95.35	230.62	16	391.77	26.12	39.94
22	135.00	85.00	5.68	186.21	286.82	39	514.23	40.83	66.40
23	124.00	83.00	4.99	118.93	301.36	12	608.95	3386.52	29.54
24	137.00	87.00	3.10	127.31	303.99	18	1238.65	85.90	33.80
25	127.00	83.00	5.08	98.76	130.52	16	375.44	92.84	85.03
26	160.00	115.00	2.80	108.11	323.71	20	641.99	92.20	115.35
27	110.00	73.00	3.72	170.29	202.01	16	735.12	53.01	51.14
28	97.00	49.00	4.00	116.82	217.02	26	423.25	52.07	106.93
29	121.00	84.00	5.60	234.74	247.45	24	383.59	68.12	83.28
30	150.00	98.00	3.57	133.77	570.53	25	857.36	39.50	25.52
31	116.00	81.00	2.13	170.37	330.07	17	533.18	187.83	31.28
32	174.00	119.00	2.43	169.73	229.81	30	566.97	30.52	45.95
33	120.00	75.00	5.54	70.63	258.49	15	319.71	24.39	35.38
34	130.00	80.00	1.65	145.54	293.12	21	352.10	99.80	75.89
35	120.00	90.00	5.16	74.89	234.72	20	377.79	361.60	56.69
36	129.00	75.00	4.30	77.99	108.86	12	293.34	199.46	92.68
37	139.00	80.00	4.94	125.21	225.85	19	904.74	45.30	117.53
38	130.00	80.00	4.55	169.72	301.15	21	550.82	25.08	68.84
39	156.00	104.00	2.62	132.34	371.92	10	640.11	239.51	30.19
40	178.00	142.00	2.31	172.10	213.97	40	1009.41	111.71	76.09
41	144.00	88.00	4.57	97.69	329.85	17	395.96	96.40	36.09
42	122.00	86.00	4.35	149.48	268.64	33	490.66	19.10	72.55
43	113.00	74.00	4.08	193.80	231.86	35	568.47	92.44	66.71
44	128.00	68.00	2.51	102.60	200.03	19	529.06	74.59	37.03
45	114.00	73.00	4.17	134.59	245.43	16	586.98	48.59	75.84
46	120.00	72.00	4.77	108.63	327.87	8	539.08	58.07	49.83

Number	TG mg/dl	Cholesterol Mg/dl	LDL mg/dl	HDL Mg/dl	Endostatin ng/ml
1	78.48	167.88	109.40	45.42	78.09
2	102.09	145.22	84.88	40.38	116.94
3	38.73	142.20	78.40	57.34	110.23
4	113.47	200.15	120.29	52.04	89.64
5	197.08	203.07	119.89	40.79	85.48
6	125.34	185.91	102.76	58.79	78.45
7	56.19	170.65	106.20	53.90	103.00
8	114.24	217.80	139.30	48.55	70.09
9	72.15	183.17	96.46	72.80	66.42
10	91.08	227.42	150.59	49.51	61.76
11	64.19	174.20	97.58	63.15	74.85
12	87.72	165.04	99.74	46.75	96.14
13	233.83	189.60	97.24	39.29	94.34
14	94.45	139.16	81.36	41.82	97.20
15	46.72	193.40	127.63	60.94	88.53
16	107.76	172.06	97.13	44.47	79.96
17	58.48	155.72	84.38	56.08	109.00
18	120.74	192.79	95.48	66.77	93.03
19	85.37	137.78	72.38	44.35	91.24
20	98.46	250.93	167.06	43.23	46.11
21	137.52	209.70	123.81	55.95	80.44
22	341.57	206.15	95.75	43.31	108.04
23	62.45	206.26	128.02	64.86	96.27
24	117.02	201.30	117.64	48.37	89.77
25	256.74	185.68	88.55	44.86	86.33
26	178.96	177.30	91.12	41.28	102.87
27	126.63	188.82	105.45	59.64	96.89
28	116.08	203.69	120.30	51.32	86.96
29	118.40	161.18	77.99	48.17	111.39
30	107.70	218.42	132.85	62.02	86.88
31	55.83	177.59	100.07	53.58	135.49
32	134.05	219.75	134.01	46.22	74.08
33	265.31	179.41	77.21	51.33	95.39
34	70.96	131.73	57.05	52.19	86.76
35	118.03	151.04	45.46	74.53	72.11
36	112.05	217.06	146.13	47.08	63.46
37	53.73	146.94	75.17	54.13	88.84
38	56.86	207.30	138.53	56.10	120.57
39	182.07	195.24	105.62	64.62	81.11
40	88.21	197.91	128.71	51.19	78.38
41	127.68	172.27	70.07	59.20	85.17
42	105.08	212.92	118.26	65.62	76.41
43	65.68	228.40	169.45	49.03	93.65
44	138.74	223.07	126.11	63.99	102.49
45	50.57	171.97	94.58	73.90	132.59
46	69.37	122.10	79.00	30.43	106.24

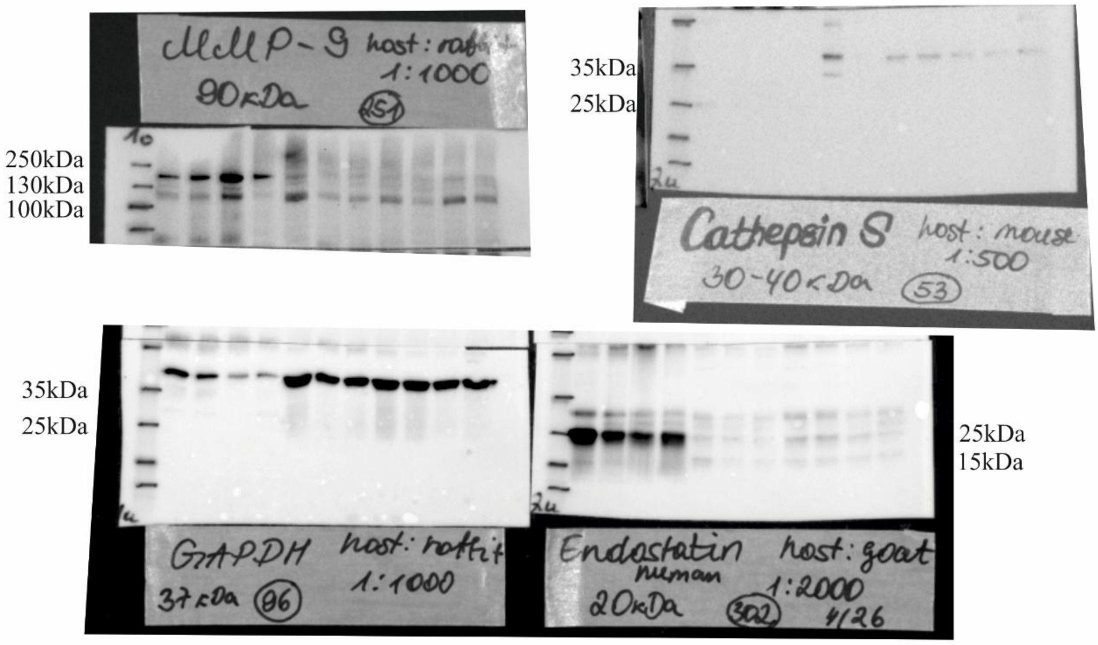

Figure S1 Original Western Blots
